# An Immobilization Technique for Long-Term Time-Lapse Imaging of Explanted *Drosophila* Tissues

**DOI:** 10.3389/fcell.2020.590094

**Published:** 2020-10-06

**Authors:** Matthew P. Bostock, Anadika R. Prasad, Rita Chaouni, Alice C. Yuen, Rita Sousa-Nunes, Marc Amoyel, Vilaiwan M. Fernandes

**Affiliations:** ^1^Department of Cell and Developmental Biology, University College London, London, United Kingdom; ^2^Centre for Developmental Neurobiology, King’s College London, London, United Kingdom

**Keywords:** *Drosophila*, live imaging, neuroblasts, adult stem cells, cell migration, cell proliferation, optic lobe, explant culturing

## Abstract

Time-lapse imaging is an essential tool to study dynamic biological processes that cannot be discerned from fixed samples alone. However, imaging cell- and tissue-level processes in intact animals poses numerous challenges if the organism is opaque and/or motile. Explant cultures of intact tissues circumvent some of these challenges, but sample drift remains a considerable obstacle. We employed a simple yet effective technique to immobilize tissues in medium-bathed agarose. We applied this technique to study multiple *Drosophila* tissues from first-instar larvae to adult stages in various orientations and with no evidence of anisotropic pressure or stress damage. Using this method, we were able to image fine features for up to 18 h and make novel observations. Specifically, we report that fibers characteristic of quiescent neuroblasts are inherited by their basal daughters during reactivation; that the lamina in the developing visual system is assembled roughly 2–3 columns at a time; that lamina glia positions are dynamic during development; and that the nuclear envelopes of adult testis cyst stem cells do not break down completely during mitosis. In all, we demonstrate that our protocol is well-suited for tissue immobilization and long-term live imaging, enabling new insights into tissue and cell dynamics in *Drosophila*.

## Introduction

Live imaging is a powerful tool to elucidate mechanistic and temporal aspects of intricate biological processes. Dynamic processes such as cell migration, protein localization, axon pathfinding and branching morphogenesis are described poorly in fixed tissue, whereas live imaging can reveal features within these processes with exquisite temporal resolution (Besson et al., 2015; Rabinovich et al., 2015; Chen et al., 2016). This approach has seen dramatic improvements with 2-photon and light sheet microscopy due to the increased depth of access and diminished phototoxicity (Huisken and Stainier, 2009; Nickerson et al., 2013; Ichikawa et al., 2014). Furthermore, developments in sample preparation for *in vivo* and *ex vivo* imaging as well as in advanced computational analyses have increased accessibility to investigations of dynamic processes (Ritsma et al., 2014; Speder and Brand, 2014; Rabinovich et al., 2015; Martin et al., 2018). Notwithstanding, a major obstacle with live imaging is sample drift, which results in a structure of interest moving out of focus. This can pose challenges to image analysis of dynamic processes.

Sample drift has been combatted by using coverslips or glass slides coated with adhesive extracellular matrix proteins such as fibronectin or collagen to physically immobilize the tissue of interest. However, these steps may exert extraneous anisotropic physical stress on the sample and affect developmental mechanisms, cause injuries to fragile tissues and therefore significantly reduce imaging time (Savoian and Rieder, 2002; Siller et al., 2005; Lerit et al., 2014; Rabinovich et al., 2015). Solutions to these problems have included placing explants in agarose wells (Rabinovich et al., 2015) but without being held in place, they still move. Although there are computational algorithms that can account for sample drift, they are often slow and can result in discontinuities between frames thus decreasing confidence in the image (Parslow et al., 2014).

Live imaging has been applied to many systems but here we focus on *Drosophila melanogaster*, whose genetic tractability makes it an outstanding model to image dynamic cellular processes. The *Drosophila* embryo was one of the earliest animal systems imaged live, due to being translucent and immobile up to late stages. Dechorionated live embryos can be imaged by gluing to a coverslip and covering with halocarbon oil to minimize dehydration (Cavey and Lecuit, 2008; Parton et al., 2010). Live imaging of the *Drosophila* embryo has been used widely to elucidate nuclear and cytoplasmic behaviors in the preblastodermal embryo (Foe and Alberts, 1983; Baker et al., 1993), epithelial adhesion during dorsal closure (Jacinto et al., 2000; Kiehart et al., 2000), germ cell migration (Sano et al., 2005), neuroblast divisions (Kaltschmidt et al., 2000) and mechanisms of salivary gland formation (Sanchez-Corrales et al., 2018) among many others. Beyond the embryo, *in vivo* live imaging becomes challenging since larvae and adults move continuously and have opaque cuticles which scatter light (Aldaz et al., 2010; Rabinovich et al., 2015; Bell, 2017). Calcium oscillations across the blood-brain barrier have been imaged through the thinner cuticle of very young larvae, reasonably steadied between coverslip and culture dish (Speder and Brand, 2014). Notwithstanding, while this methodology was apt for capturing relatively large-scale inter-cellular calcium wave propagation, the considerable drift remaining is not suited to visualize finer (sub)cellular events. Similarly, although larvae and pupae have been imaged live, the need to strike a balance between phototoxicity and image-acquisition rates often mean that some dynamic processes are hard to capture (Bosveld et al., 2012; Ghannad-Rezaie et al., 2012; Heemskerk et al., 2014; Tsao et al., 2016; Dye et al., 2017). In adults, live imaging can be performed through windows cut out of the cuticles of immobilized animals (Fiala et al., 2002; Seelig et al., 2010; Martin et al., 2018; Aimon et al., 2019) but feasibility of this approach depends on the accessibility of the tissue of interest.

An alternative to *in vivo* imaging is to image tissues in culture. Initially, explanted tissues were imaged to study processes over short periods of time (i.e., minutes to hours) such as cell cycle progression and oriented cell divisions, epithelial cell packing, intracellular protein movements and secretion (Siller et al., 2005; Farhadifar et al., 2007; Siller and Doe, 2008; Aldaz et al., 2010; Mao et al., 2011; Lerit et al., 2014). More recently, live imaging of cultured explants has been extended to processes that unfold over several hours such as morphogenesis of pigment cells during pupal eye development (Hellerman et al., 2015), cell migration (Prasad et al., 2015; Chen et al., 2016; Barlan et al., 2017), neuronal remodeling (Rabinovich et al., 2015), growth cone dynamics (Ozel et al., 2015; Akin and Zipursky, 2016), and spermatogonial stem cell dynamics in their niche (Sheng and Matunis, 2011; Lenhart and DiNardo, 2015).

Different culture media compositions have been applied to long-term live imaging of explanted *Drosophila* tissues. The most commonly used is Schneider’s Insect medium (Echalier, 1997). Echalier’s D-22 medium (Siller et al., 2005; Lee et al., 2006), Shield’s and Sang’s M3 medium (Aldaz et al., 2010) and Grace’s Insect Culture medium have also been employed (Dye et al., 2017). These media are often supplemented with exogenous growth supporting components such as insulin, fetal bovine serum, fly extract, larval fat bodies, ascorbic acid and/or 20-hydroxy-ecdysone (20E) to optimize culture conditions. Supplement requirements vary with the tissues being imaged (Parton et al., 2010) and there are conflicting opinions regarding supplements for the same tissue. For example, some studies report that the addition of fly extract is essential to support imaginal disc growth *ex vivo* (Wyss, 1982; Zartman et al., 2013; Restrepo et al., 2016) whereas others demonstrated that fly extract had no effect on disc growth and in fact caused aberrant calcium oscillations in cultured wing discs (Tsao et al., 2016; Balaji et al., 2017). Similarly, larval fat bodies were found to be vital to maintain neuroblast divisions *ex vivo* (Siller et al., 2005; Cabernard and Doe, 2013) but others either found them dispensable for neural proliferation in the young larval central nervous system (CNS) or inhibitory of early pupal CNS development (Sousa-Nunes et al., 2011; Rabinovich et al., 2015). Lastly, addition of 20E and insulin to culture medium aimed at supporting imaginal disc growth has also been debated. Absence of insulin and presence of 20E has been reported to enable disc growth *ex vivo* (Aldaz et al., 2010; Dye et al., 2017) although other studies suggest that insulin is necessary (Restrepo et al., 2016; Tsao et al., 2016) but that 20E impairs disc development (Tsao et al., 2016). Differences in tissue responses to these supplements might be attributed to the specific stage and/or basal medium being used. For example, Zartman et al. (2013) demonstrated that cells derived from wing discs proliferated to a greater extent when insulin was added to Schneider’s Insect Medium but not M3 medium (Zartman et al., 2013).

Here, we present a simple protocol for culturing and imaging *Drosophila* larval and adult tissues *ex vivo*. We use Schneider’s Insect Medium along with relatively few growth supplements and immobilize samples in low gelling temperature agarose, an adaptation of the method commonly used to immobilize zebrafish embryos or larvae for live imaging (Distel and Koster, 2007). In this way, the explanted tissue is held in place without imposing anisotropic physical stress on it. Moreover, this technique allows tissues to be held in any orientation, independent of shape and center of gravity, rendering imaging of fine features readily accessible. A similar agarose-based immobilization technique was recently described for short-term imaging of larval neuroblast divisions (Miszczak and Egger, 2020). We have used this approach successfully to image the migration of glial cells and neurons in the *Drosophila* brain during the third larval instar over long developmental periods (Chen et al., 2016; Rossi and Fernandes, 2018). Here, we validate our protocol in multiple tissues from multiple developmental stages and report new biological observations for the first time. Specifically, we followed neuroblast divisions not only in the commonly-imaged wandering third larval instar (wL3) brain but also as they reactivate from quiescence during the first and second larval instars (L1 and L2); we captured glial and neuronal migration in the optic lobe, assembly of lamina columns and eye-antennal disc eversion; and we imaged cyst stem cell mitoses in adult testes. Overall, this is an inexpensive and simple method to carry out live imaging experiments to broaden understanding of cell and tissue dynamics in *Drosophila*.

## Materials and Methods

See supplementary material for a detailed step-by-step protocol with suggested volumes.

### Fly Husbandry and Stocks

Fly strains and crosses were raised on standard cornmeal food at 25°C, except for the sparse labeling of epithelial and marginal glia (*dpp > FlexAmp*), which was raised at 29°C.

The Following genotypes were used in this study: *{yw; gcm-GAL4/CyO;}* Bloomington Drosophila Stock Center (BDSC) #3554, *{;; UAS-CD8::GFP/TM6B}* BDSC #5130, *{;; UAS-nls::GFP/TM6B}* BDSC #4776, *{; 13xLexAop-6xmcherry/CyO;}* BDSC #52271, *{yw, UAS-FLP; GAL80^*ts*^/CyO; Act > y+ > lexA, lexAop-myr::GFP/TM6B} (FlexAmp)* (Bertet et al., 2014), *{;; dpp-GAL4/TM6B}* BDSC #7007, *{; E-Cad-E-Cad::GFP;}* (Huang et al., 2009), *{;; R27G05-LexA/TM6C}* (Tan et al., 2015), *{; ubi-GFP::CAAX;}* Drosophila Genomics Resource Center (DGRC) *#109830, {; His2av::EGFP/SM6a;}* BDSC #24163, *{; Tj-GAL4;}* DGRC #104055, *{; grh-GAL4;}* (Chell and Brand, 2010), *{;; UAS-syn21-GFP-p10}* (Pfeiffer et al., 2012), *{;; UAS-CD4-tdTomato}* a gift from D, Williams.

### Explant Culture Medium

Explant culture medium consisted of Schneider’s Insect medium (Sigma #S0146) supplemented with 2.5 μL/mL human insulin (Sigma #I9278), 1 % Penicillin-Streptomycin (Sigma #P4333) and 10 % Fetal Bovine Serum (Sigma #F2442) stored at 4°C and used within a month of preparation.

### Preparation of Low Gelling Temperature Agarose

A total 2% low-gelling temperature agarose (Sigma #A9414) was prepared in sterile water. These were cut into ∼0.5 cm^3^ pieces and stored in distilled water at 4°C. The agarose was deionized by changing the water each day for 5 days before use.

### Dissections

Forceps, dissection pads, pipettes, falcon tubes and working areas were wiped down with 70% ethanol before use. Dissection of L1, L3 and adult tissues was carried out in cold culture medium. Dissections of L1 CNSs were performed using forceps to hold down the posterior end of the larva and a tungsten needle to slowly rip open the larval cuticle, and then lightly pull on the mouth hooks to extract the CNS. CNSs were left attached to mouth hooks via the esophagus, as well as to surrounding fat tissue and imaginal discs to avoid damage. Dissections of L3 CNSs were performed with a pair of forceps, used to rip and remove the larval cuticle, and sever the CNS from the midgut. Fat tissue and imaginal discs were removed, leaving only mouth hooks attached to the CNS via the esophagus. For L1–L3 CNS imaging esophageal muscles were crushed to cease unwanted contractions. For adult testes, flies were dissected 0–3 days post-eclosion with careful removal of the ejaculatory duct and accessory glands of the male gonad leaving each testis intact with its connecting seminal vesicle.

### Tissue Immobilization in Agarose

Deionized agarose (see above) was melted in a microwave for approximately 20–30 s (per 0.5 cm^3^ cube of 2% low-gelling temperature agarose) and diluted to 0.4% in culture medium heated to 42°C using a programmable heating block. The temperature was then lowered to 34°C before being added to coat the bottom of untreated 35 × 10 mm petri dishes (Thermo #171099). A single explant was placed in each dish and maneuvered to the center to be oriented using forceps. To maneuver the tissue, forceps were used to move the viscous agarose rather than the tissue itself. Once the desired orientation was achieved, the forceps were gently withdrawn from the agarose, which held the tissue in place due to its viscosity. All movements and orientations of the tissue were achieved within 5 min of placing the brain in the agarose so as not to disrupt its setting. The agarose was left to solidify for 10 min after which cold culture medium was added.

### Imaging and Image Processing

We used an upright microscope set-up (Olympus FV1000MPE multi-photon laser scanning microscope or Zeiss 880, both with Spectra-Physics Mai Tai DeepSee 2-photon lasers) with water-immersion lens (Olympus XLPLN 25X WMP2 or Zeiss Plan-Apochromat 20X). The objective was immersed directly in the culture medium for imaging. The fluorophores used were all GFP or RFP derivatives, therefore the excitation wavelength was tuned between 925 and 935 nm. Laser power never exceeded 15%. Zen Blue (Zeiss) and ImageJ software was used to analyze movies. The Bleach Correction and Manual Tracking plugins were used to correct photobleaching and to track cells. The Correct 3D Drift plugin was used to correct for movements caused by tissue contraction. Adobe Photoshop (v21.1.3) and Adobe Premiere Pro (v14.2) were used to annotate and edit movies. Figures were generated using Adobe Illustrator (v24.1.3).

## Results

### Neuroblast Divisions in the L3 Central Brain

**FIGURE 1:**
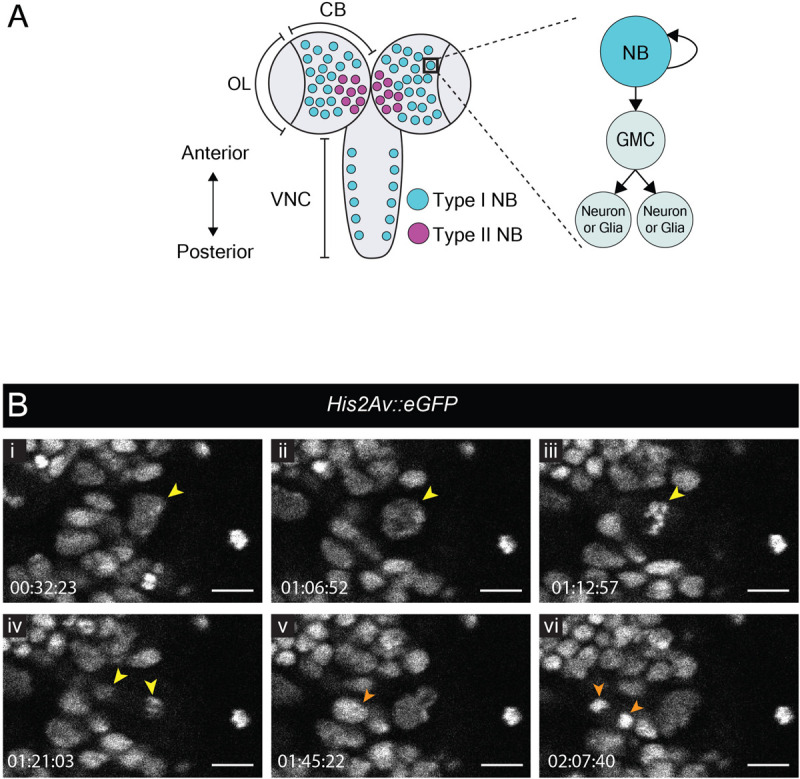
Neuroblast and GMC divisions in the central brain during L3. **(A)** A schematic of the dorsal view of an L3 CNS, which is made up of the optic lobes (OL), the central brain (CB) and the ventral nerve cord (VNC) (Sousa-Nunes and Hirth, 2016). Type I neuroblasts (blue), which divide to self-renew and generate a GMC, are most abundant and are present throughout the CNS. GMCs divide symmetrically in size to generate two differentiating neuronal or glial progeny. Type II neuroblasts (magenta), defined by generation of two types of transit-amplifying progenitors (intermediate neural precursors and GMCs) consist of eight paired lineages found in the dorsoposterior regions of the CB. The black box indicates the region selected for live imaging, which contains only type I neuroblasts. **(B)** A time-series extracted from Movie 1 showing a cultured *His2aV::eGFP* brain (wL3) with a Type I neuroblast nucleus (yellow arrowhead) and a GMC nucleus (orange arrowhead) undergoing cell divisions. Timescale displayed as hh:mm:ss, scale bar = 10 μm.

The late larval CNS has been used extensively to study the biology of neural stem cells, called neuroblasts in *Drosophila*. Neuroblasts generate a vast number of diverse neuronal and glial cell subtypes which are critical for neural function. So-called type I neuroblasts are the most abundant and are found throughout the CNS (Figure 1A). They divide asymmetrically to self-renew and generate a transit-amplifying progenitor called ganglion mother cell (GMC) [reviewed by Sousa-Nunes and Hirth (2016)]. The GMC then undergoes a terminal division to produce two neuronal and/or glial progeny whereas the self-renewed neuroblast continues to proliferate. To compare our protocol to existing strategies for visualizing neuroblast dynamics, we imaged divisions in the central brain from animals dissected at the wL3 stage (Figure 1). To visualize chromatin we used *His2Av::eGFP*, a histone variant fused to an enhanced green fluorescent protein (eGFP); larval neuroblasts were identified as large (∼10–15 μm diameter) superficial cells (Truman and Bate, 1988). As expected, these cells underwent a self-renewing division to generate a neuroblast and a GMC (Figure 1B and Movie 1). Between divisions, neuroblasts grew in size and their cell-cycle time was ∼90 min, consistent with other reports (Cabernard and Doe, 2013; Homem et al., 2013). We also observed GMC divisions (Figure 1B and Movie 1). Although it has been reported that larval fat bodies are essential for sustaining neuroblast divisions *ex vivo* (Siller et al., 2005; Cabernard and Doe, 2013), we found them to be dispensable here. In summary, neural progenitor divisions proceeded as expected, demonstrating that our culture medium and immobilization technique can support them.

### Neuroblast Reactivation

**FIGURE 2:**
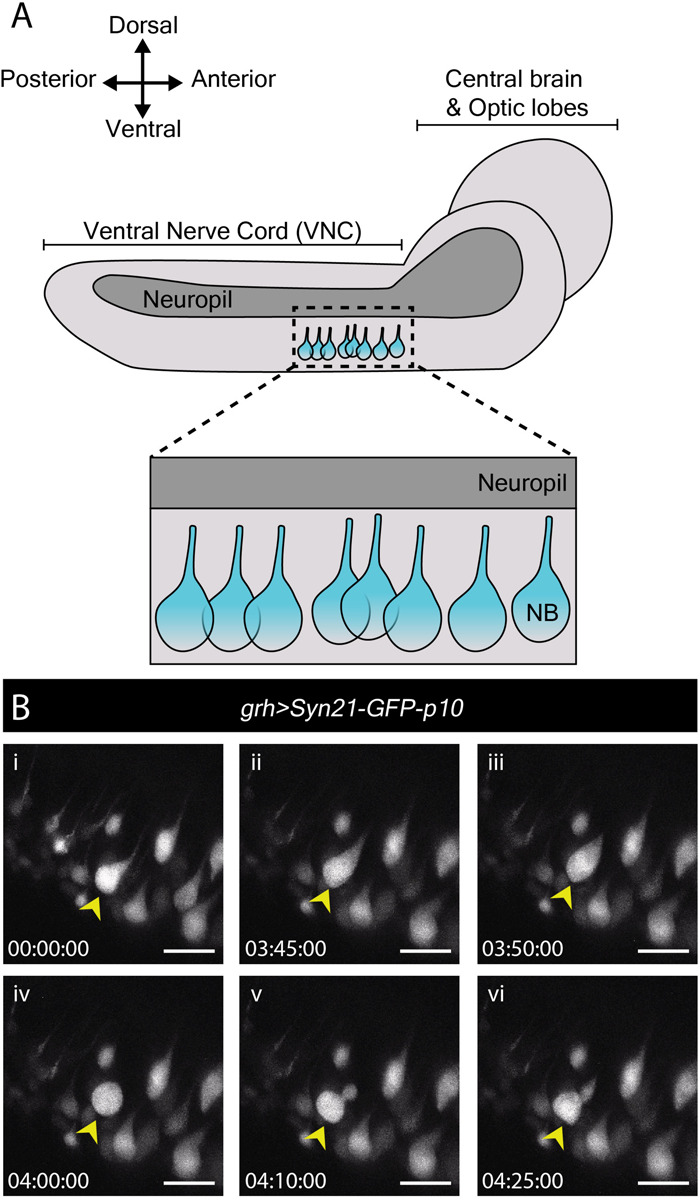
Reactivation of quiescent neuroblasts in the L1/L2 CNS. **(A)** A schematic of the lateral view of an L1/L2 CNS. Neuroblasts, depicted in blue, are shown within the thoracic region of the ventral nerve chord (VNC, dashed box), the region selected for live imaging. Neuroblast (NB) cell bodies are situated on the ventral side of the VNC. During quiescence, they extend a fiber toward the neuropil. Upon reactivation, which occurs roughly 24 h after larval hatching for neuroblasts in the thoracic VNC, neuroblasts lose their fiber, which marks a relatively early morphogenetic event we sought to capture through our live imaging protocol. **(B)** A time-series extracted from Movie 2 of a cultured *grh > Syn21-GFP-p10* brain (L1/L2) showing an example neuroblast in the VNC (yellow arrowhead), which reactivates first by moving dorsally, then rounding before undergoing an asymmetric division to produce a dorsally positioned GMC. Timescale displayed as hh:mm:ss, scale bar = 10 μm.

In contrast to many studies employing live imaging of neuroblasts from L3 CNSs, none as yet report imaging of neuroblast divisions in the more fragile first or second larval instars (L1/L2). Nonetheless, these earlier stages include specific processes of interest. By the end of embryogenesis, most neuroblasts enter a state of reversible cell cycle arrest termed quiescence (Truman and Bate, 1988; Valcourt et al., 2012). Following larval hatching and feeding, neuroblasts exit quiescence (reactivate) in an anteroposterior order (Truman and Bate, 1988; Britton and Edgar, 1998; Chell and Brand, 2010; Sousa-Nunes et al., 2011). Neuroblasts are relatively large when actively proliferating (10–15 μm diameter) (Chell and Brand, 2010; Sousa-Nunes et al., 2011) and are devoid of morphological polarity despite extensive molecular asymmetries during mitosis. In contrast, quiescent neuroblasts are much smaller (∼4 μm diameter) and morphologically polarized, projecting a basal fiber into the neuropil (Figure 2A). This morphology is reminiscent of that of vertebrate radial glia (Weissman et al., 2003) and renders quiescent neuroblasts morphologically indistinguishable from adjacent neurons (Figure 2A). As they reactivate, neuroblasts enlarge and lose their fiber. We wondered whether fibers would be severed or retracted during neuroblast reactivation and endeavored to image this process live.

Young larval CNSs are more susceptible to mechanical stress than later ones, including to forces exerted by laminin or poly-L-lysine-coated surfaces (our own observations). Immobilizing L1 or L2 brains in this way invariably resulted in CNS rupture. While L1 and L2 CNSs did not rupture when immobilized under a fibrin clot (Lerit et al., 2014), we were unable to orient them at will to visualize neuroblasts clearly using this method (data not shown). The agarose-based immobilization approach described here proved sufficiently gentle and allowed for the desired orientation, enabling documentation of neuroblast reactivation for the first time.

To reactivate, neuroblasts require a fat body signal or downstream glial-derived Insulin-like peptides, produced in response to larval feeding (Britton and Edgar, 1998; Chell and Brand, 2010; Sousa-Nunes et al., 2011). Therefore, we imaged neuroblast reactivation in the ventral nerve cord of CNSs dissected at 22–24 h after larval hatching (late L1/early L2), in which nutritional-dependent signals are already present (reported by soma enlargement and EdU incorporation in the brain lobes (Britton and Edgar, 1998; Chell and Brand, 2010; Sousa-Nunes et al., 2011). *grainyhead (grh)-GAL4*, expressed in a subset of neuroblasts (Chell and Brand, 2010) was used to drive expression of *UAS-Syn21-GFP-p10*, a translationally enhanced GFP reporter (Pfeiffer et al., 2012). Several ventral nerve cord neuroblasts were observed reactivating and undergoing mitosis over the course of 17 h (Figure 2B and Movie 2). Mitoses were clearly recognizable by cells rounding prior to dividing into one larger apical daughter (renewed neuroblast) and one smaller basal daughter (the GMC). Neuroblast and GMC divisions continued after reactivation in a few cases (*n* = 6) indicating favorable conditions.

To our surprise, we found that neuroblasts retained their fiber throughout the first post-reactivation division and that the fiber was inherited by the first post-reactivation GMC (Movie 3, Part 1; *n* = 19). Asymmetric basal fiber inheritance has been described for zebrafish, rodent and human embryonic/fetal neural progenitors. Intriguingly, in contrast to what we observed in *Drosophila*, in those models it was generally the self-renewing progenitor that inherited the fiber (Weissman et al., 2003; Konno et al., 2008; Alexandre et al., 2010; Hansen et al., 2010; Shitamukai et al., 2011) although, on occasion, asymmetric inheritance by neuronal progeny was observed (Miyata et al., 2001; Konno et al., 2008), as was fiber splitting and seemingly symmetric inheritance by both daughter cells (Konno et al., 2008). In the few cases where we were able to follow the basal fiber throughout a GMC division (*n* = 5), the fiber appeared to be inherited by GMC progeny (Movie 3, Part 2). Further work is necessary to assess the generality of this finding, but we speculate that fiber inheritance by GMCs and then neuronal progeny could be a mechanism to develop neurites quickly, especially important for a fast-developing organism like *Drosophila*.

The above demonstrates that our protocol is well-suited to immobilize early larval brains even in the generally unstable side orientation for long-term neuroblast imaging, including observation of the first post-reactivation division and GMC fiber inheritance from quiescent neuroblasts, which has not been reported before.

### Lamina Development in the L3 Optic Lobes

**FIGURE 3:**
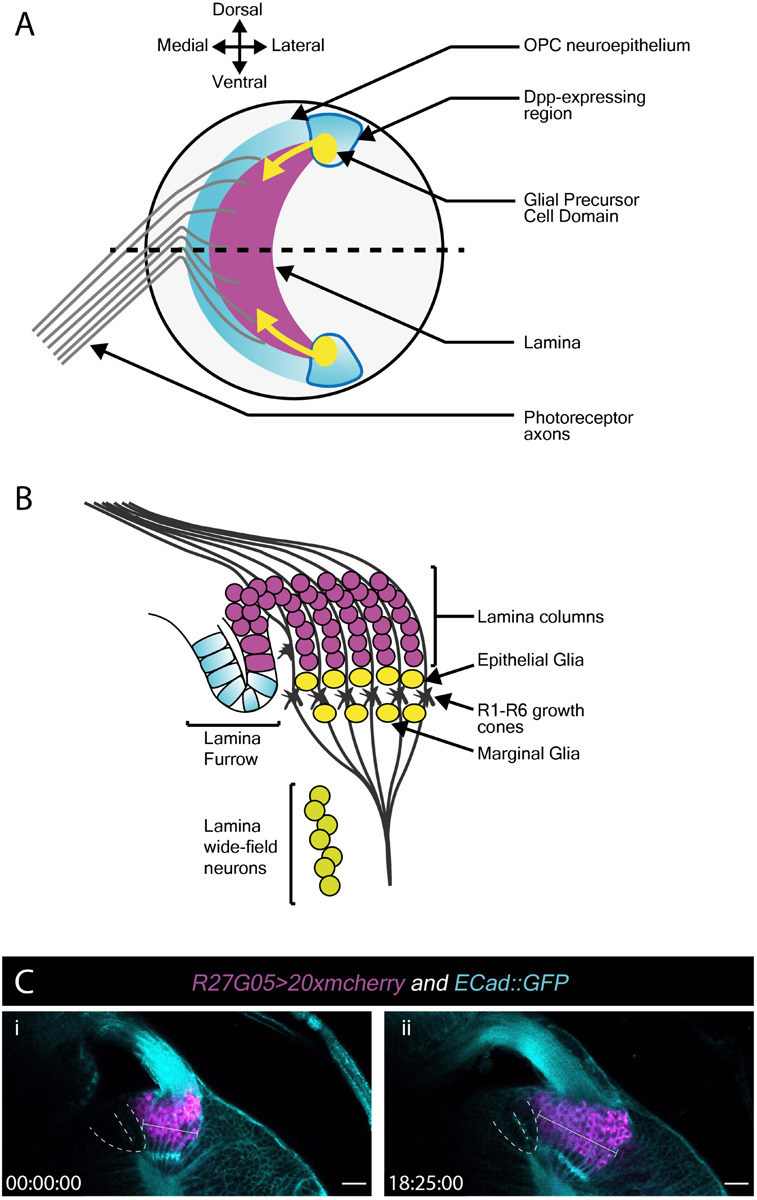
Lamina development including the migration of epithelial and marginal glia, and lamina wide-field neurons (Lawf)**. (A)** Schematic of the lateral view of the third larval instar optic lobe. Developing photoreceptors project to a fold in the outer proliferation center neuroepithelium called the lamina furrow and induce lamina formation. **(B)** Diagram of a cross-section along the dotted line in A. The developing lamina forms in characteristic columns. Seven lamina precursor cells are incorporated into each column. Epithelial and marginal glia migrate above and below photoreceptor growth cones. Lawf neurons share the same progenitors as epithelial and marginal glia; they migrate from their point of origin at the tips of the lamina to the medulla, where they stop immediately adjacent to the neuropil. **(C)** Two timepoints extracted from Movie 3 of a cultured *R27G05 > 20xmcherry* (magenta) and *Ecad::GFP* (cyan) brain (wL3) showing the lamina, photoreceptor axons and surrounding tissue. The lamina furrow is marked by a dashed line. The lamina grows considerably over ∼18 h as indicated by the bracket. Timescale displayed as hh:mm:ss, scale bar = 20 μm.

Next, we turned our attention to the developing L3 optic lobe, specifically focusing on the developing lamina. The lamina arises from a crescent-shaped neuroepithelium called the outer proliferation center (OPC), which is located at the surface of the optic lobe. The lateral edge of the OPC folds to form a structure called the lamina furrow (LF), from which lamina precursor cells (LPCs) are generated (Figures 3A,B). Photoreceptors from the eye disc grow their axons through the optic stalk and into the optic lobe where they defasciculate and contact the LF along the dorsoventral length of the OPC crescent. R1–R6 photoreceptors terminate their growth cones at the level of the LF (Figure 3B). Photoreceptors deliver Hedgehog through their axons and induce LPC formation from LF neuroepithelial cells (Figure 3A; Huang and Kunes, 1996; Huang et al., 1998). LPCs then associate with photoreceptor axons to form columns before differentiating into lamina neurons (Huang and Kunes, 1996; Huang et al., 1998; Umetsu et al., 2006; Fernandes et al., 2017).

To visualize lamina development we used *E-Cadherin::GFP* (*E-Cad::GFP*), which localizes to epithelial adherens junctions, together with the lamina-specific (*R27G05-GAL4*) expression of cytoplasmic *mCherry* (Figure 3C and Movie 4). Photoreceptor axons and the lamina furrow showed enriched *E-Cad::GFP* expression (Figure 3C and Movie 4). The lamina grew dramatically over the course of ∼18 h (Figure 3C and Movie 4). Interestingly, throughout this growth the lamina furrow remained relatively stable (Figure 3C and Movie 4 – asterisk), with lamina growth displacing older lamina columns posteriorly (Movie 4). This is in contrast to previous assumptions based on fixed images that the lamina furrow moved similarly to the morphogenetic furrow in the eye imaginal disc (Selleck and Steller, 1991; Huang and Kunes, 1998) and implies a different process by which LPCs are generated from the neuroepithelium.

The use of cytoplasmic mCherry prevented us from distinguishing individual LPCs and their incorporation into columns. We therefore switched to nuclear GFP (*UAS-nlsGFP*) driven by *glial cells missing (gcm)-GAL4*, which marks LPCs and lamina glia (Figures 3A,B and Movie 5). We manually tracked LPCs as they exited the LF through to incorporation into columns. Rather than column assembly progressing one column at a time, we observed that LPCs incorporated into the first 2–3 columns simultaneously, suggesting that multiple young columns are assembled together (Movie 5). This was surprising since it was generally assumed that the lamina is built one row of columns at a time (Umetsu et al., 2006; Sugie et al., 2010; Sato et al., 2013).

### Glial and Neuronal Migration in the L3 Optic Lobes

In addition to LPCs, the developing lamina is also populated by glia. Epithelial and marginal glia are positioned above and below photoreceptor growth cones (Figure 3A). These glia originate from glial precursor cell domains at the dorsal and ventral tips of the lamina and migrate tangentially into the developing lamina (Figure 3A; Dearborn, 2004; Yoshida et al., 2005; Chen et al., 2016). When viewed in cross-section (Movie 5), we noticed that epithelial glia, situated above the photoreceptor growth cones (Figure 3A), were very motile and moved across photoreceptor growth cones and sometimes below to the level of marginal glia (Movie 5). Though they originate from the same domains, epithelial and marginal glia are distinct cell types (Chotard and Salecker, 2007; Edwards and Meinertzhagen, 2010; Edwards et al., 2012). While they express different molecular markers at later developmental stages, at L3 they have been distinguished solely by their by their relative positions on either side of photoreceptor growth cones (in fixed samples) (Chotard and Salecker, 2007; Edwards et al., 2012). In our L3 live imaging, lamina glial positions were not as stable as expected from previous descriptions (Movie 5). We also observed glial migration toward the anterior side of the lamina from posterior positions (Movie 5), most likely a consequence of glial incorporation into young lamina columns.

Epithelial and marginal glia have neuronal siblings, which develop into two neuron subtypes called lamina wide-field neurons 1 and 2 (Lawfs) (Chen et al., 2016; Suzuki et al., 2016). Lawfs are also born in the glial precursor cell domains and migrate tangentially but below the level of glia to incorporate into the deepest layers of the medulla (Chen et al., 2016). Since *gcm-GAL4* labels Lawfs as well as LPCs and lamina glia, we used it to express membrane-tagged GFP (*UAS-CD8::GFP*) to visualize glial and Lawf neuronal migration (Movie 6). Lawf migration was readily captured as described (Chen et al., 2016). However, the dense packing of labeled glia proved challenging for tracking these cells (Figure 3A and Movie 6). We therefore switched to a sparse labeling technique, called FlexAmp (Bertet et al., 2014) to induce stochastic and permanent expression of *myristoylated-GFP (myr-GFP)* in the glial precursor cell domains and thus progeny originating therein. Using *dpp-GAL4* to induce sparse labeling, GFP-positive cells were observed at the dorsal and ventral tips of the lamina and in the lobula plug, where *dpp-GAL4* is expressed (Figure 3A and Movie 7). Furthermore, we could clearly track the migration of several glia originating from these domains into the lamina. These glia displayed many dynamic membrane protrusions (Movie 7), as inferred by others from fixed tissue (Poeck et al., 2001; Yoshida et al., 2005). Overall, we show that our live imaging protocol can be used to capture dynamic processes involved in cell migration during lamina development. We revealed cell behaviors that were not apparent from fixed tissue, including membrane protrusion dynamics during glial migration and epithelial and marginal glial incorporation into lamina columns, suggesting that these two glial cell types are not strictly separate until later in development.

### Eye Disc Eversion

**FIGURE 4:**
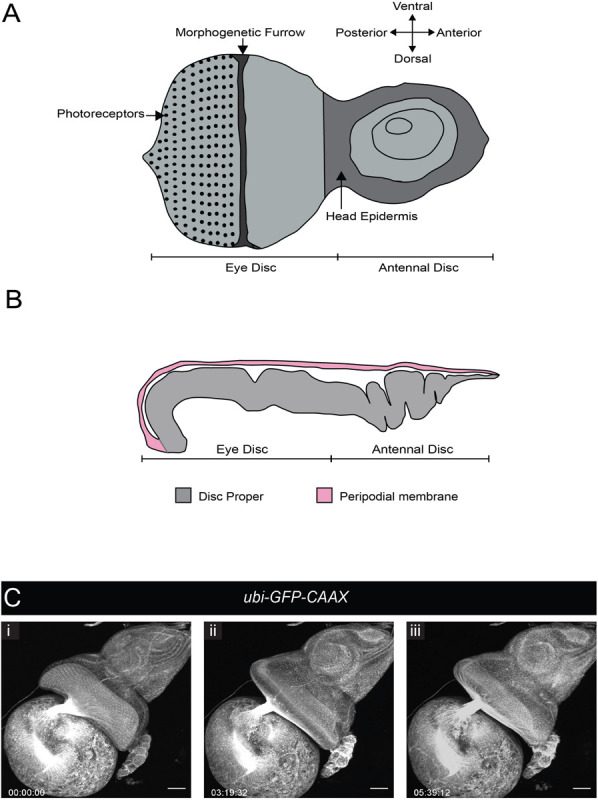
Eye-antennal disc structure and disc eversion. **(A)** Schematic of an L3 eye-antennal disc. **(B)** Cross-section of the eye-antennal disc. The columnar epithelium of the disc proper at the anterior end (antennal side) is folded whereas that of the eye disc is stretched and convex in shape. The thin peripodial membrane sits above and is continuous with the disc proper which constitutes the eye disc and the antennal disc. **(C)** A time-series extracted from Movie 8 (maximum intensity projection) of a cultured *ubi-GFP-CAAX* eye-antennal disc-brain complex (wL3) showing the eye-antennal disc undergoing eversion. Timescale displayed as hr:mm:ss, scale bar = 40 μm.

During wL3 stage, the eye-antennal discs (EADs) undergo complex remodeling to give rise to several adult head structures and the head epidermis (reviewed by Kumar, 2018). One of the most prominent metamorphic events that primes the EADs to generate their corresponding adult appendages is disc evagination, which is subdivided into two discrete processes – elongation and eversion (reviewed by Gibson and Schubiger, 2001). The EADs are comprised of two epithelial layers, the columnar disc proper and a squamous epithelium called peripodial membrane. The peripodial membrane sits atop and is continuous with the disc proper (Figure 4), with interaction between the two described as vital for disc eversion (Milner et al., 1983). To date, studies focused on this dynamic event have been limited to fixed tissues (Gibson and Schubiger, 2001), largely attributed to absence of appropriate culturing and immobilization systems given anisotropic forces exerted by biological glues that likely affect morphogenesis (Kumar, 2018).

We tested whether our culture system and immobilization technique could be used to capture EAD eversion. We dissected late L3 CNSs ubiquitously expressing a membrane-bound GFP (*ubi-GFP-CAAX*) with attached EADs. These explanted EADs immobilized in agarose underwent disc eversion within a period of 5 h (Figure 4C and Movie 8). The peripodial membrane appeared to contract and pull the eye disc proper toward the larval epidermis. The eye disc curled anteriorly taking on an oval shape, at the same time the antennal disc was molded into a circular shape. This morphological change of the antennal discs is important to drive their movement outside the larval epidermis and then fusion to form the adult head epidermis (Milner et al., 1984). Since our culture system and immobilization technique recapitulated disc eversion events as observed in histological studies of cultured EADs carried out by others (Milner et al., 1983), it can be used to study and visualize in real time how the peripodial membrane affects disc eversion. For example, EADs in which the peripodial membrane is genetically or physically ablated can be imaged live to further analyze the mechanics of disc eversion.

### Stem Cell Maintenance in the Adult Testis

**FIGURE 5:**
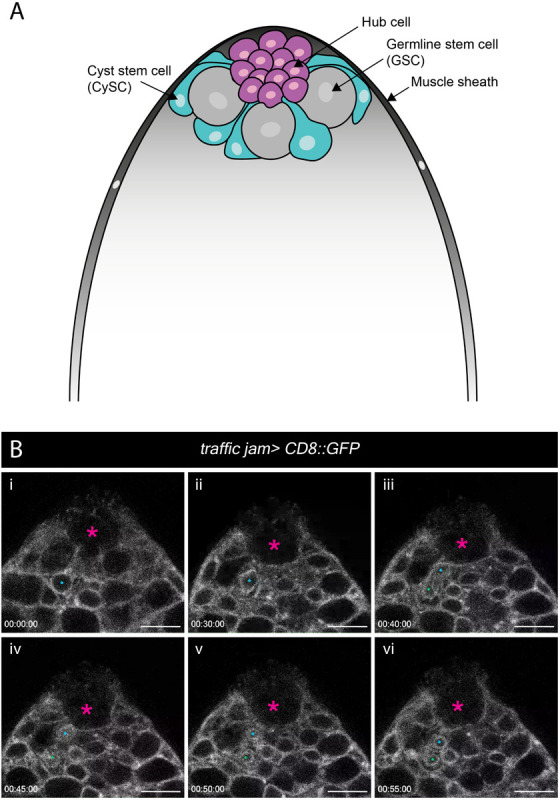
Spatial organization of stem and niche cells in the adult testis and mitosis in the CySCs. **(A)** Schematic of the adult testis, which is encased in a muscle sheath (dark gray). Anchored at the apical tip of the tissue is a cluster of small post-mitotic cells known as the hub (magenta), which constitutes the stem cell niche. Two stem cell populations are in direct contact with the hub – germline stem cells (GSCs, gray) and cyst stem cells (CySCs, cyan). GSCs are large, round cells that closely associate with the hub. CySCs are located behind GSCs and extend thin membrane protrusions between GSCs to contact the hub. **(B)** A time-series extracted from Movie 9 of a cultured *traffic jam > CD8::GFP* adult testis. The hub is marked with a magenta asterisk. A CySC that undergoes a division is marked with a blue dot (i, ii). The nuclear membrane is visible during the division and takes on a characteristic diamond shape (ii) before the two daughter cells (blue and green dots; iii, vi) pull apart. Timescale displayed as hr:mm:ss, scale bar = 10 μm.

*Ex vivo* imaging can bypass many of the technical challenges associated with imaging adult tissues such as opaque cuticle and animal movement. To test whether our *ex vivo* imaging setup could be applied to adult tissue we focused on the *Drosophila* testis, a well-characterized model to study homeostatic mechanisms regulating stem cell behaviors in their intact microenvironment. The testis stem cell niche is composed of a cluster of quiescent stromal cells collectively known as the hub (Hardy et al., 1979). The hub is anchored at the apex of a blunt-ended coiled tube that forms the testis (Figure 5A). Hub cells support two stem cell populations, germline stem cells (GSCs) and somatic cyst stem cells (CySCs). GSCs and CySCs are physically attached to the hub, and their self-renewal is maintained by hub-derived signals (Figure 5A) (reviewed by Greenspan et al., 2015). The two stem cell populations are easily distinguishable by morphology and position. GSCs are large and round cells that tightly associate with the hub whereas CySC have smaller nuclei located behind GSCs and extend a thin membrane projection between GSCs to contact the hub (Hardy et al., 1979; Figure 5A).

Previous efforts to image the testis typically involved weighing down the tissue onto the culture dish using cellulose membranes, teflon sheets or coverslips (Cheng and Hunt, 2009; Sheng and Matunis, 2011; Inaba et al., 2015) or adhering the tissue to poly-L-lysine-coated coverslips (Lenhart and DiNardo, 2015; Greenspan and Matunis, 2017). These methods exert physical stress on the tissue during imaging. Here, we used our immobilization strategy to eliminate any non-specific effects of anisotropic forces exerted on the tissue. We used the somatic lineage-specific driver, *traffic jam (tj)-GAL4* to drive membrane targeted GFP (*UAS-CD8::GFP*), thus labeling the entire lineage including the CySCs and their progeny. As documented by others previously, the muscle sheath encasing the testis can cause contractility. Testes vary in contractility, and only testes with mild movements were chosen for imaging (Sheng and Matunis, 2011; Inaba et al., 2015). Post-acquisition computational drift correction (Correct 3D Drift Plugin, ImageJ – see Methods) was sufficient to generate a stable movie for analysis for testes that displayed minor contractility. We observed multiple CySC divisions (Figure 5B and Movie 9), during which CySC nuclei moved closer to the hub and rounded up. Unexpectedly, nuclear membrane labeling by CD8::GFP was apparent and persisted throughout CySC divisions, and we observed that this nuclear labeling could be reliably used to identify dividing CySCs (Figure 5B, and Movies 9, 10). This observation suggests that CySCs divide with a closed or semi-closed nuclear division as has been reported in other *Drosophila* cells including embryos, neuroblasts and germ cell meiosis (Church and Lin, 1982; Stafstrom and Staehelin, 1984; Debec and Marcaillou, 1997; Cheng et al., 2011; Boettcher and Barral, 2013; Roubinet et al., 2020). Recent work in neuroblasts implicates asymmetric nuclear division in the control of daughter cell fates (Roubinet et al., 2020), raising the question of whether a similar mechanism may control cell fates in the testis. This work demonstrates that our protocol can be adjusted to track cellular behaviors in adult tissues.

## Conclusion

Live imaging enables novel insights into dynamic biological processes of different scales, from the subcellular to the multicellular. Here we detail a simple and inexpensive protocol for immobilizing explanted tissues in any precise orientation desired, which supports long-term live imaging with minimal physical stress. We validated our approach by visualizing dynamic processes previously described for L3 brains and adult testes and applied it to make novel observations: (1) multiple lamina columns undergo assembly together, (2) dynamic membrane protrusions and extensions of epithelial and marginal glia during migration, (3) fibers of quiescent neuroblasts are inherited by the GMC upon reactivation, and (4) the nuclear membrane of adult CySCs does not break down completely during mitosis.

Our protocol is amenable to customization with minimal effort and could be used for experimental approaches requiring temperature shifts (e.g., 29°C for temperature-sensitive mutants or increased GAL4 activity), for approaches requiring acute drug treatment by combination with a flow perfusion apparatus (Williamson and Hiesinger, 2010), and to other species.

We note that this protocol is optimized for upright microscopes using a water-dipping objective. The upright set-up is useful for certain tissue orientations, but the protocol could be used with an inverted microscope using a glass-bottomed petri dish or a live cell chamber (Miszczak and Egger, 2020). When working with an inverted microscope set-up, users should consider the objective working distance and the volume of agarose for immobilization, however. While we have not tested our protocol with an inverted set-up, the volume of agarose used to immobilize the tissue may need to be reduced or the sample pushed closer to bottom to ensure close proximity to the glass bottom for imaging.

Overall, this protocol offers a simple, inexpensive and versatile method to immobilize explanted tissues for long-term live imaging.

## Data Availability Statement

All datasets presented in this study are included in the article/Supplementary Material.

## Author Contributions

MB and AP designed and performed L3 experiments with *gcm-GAL4* and *dpp-GAL4* (MB), and *ubi-GFP-CAAX* (AP). MB analyzed the data, prepared the figures and movies, and wrote the majority of the manuscript together with AP. RC performed the L1 experiments, analyzed the data, and prepared the figures and movies. RC and RS-N wrote the corresponding text. AY performed experiments related to the adult testis, analyzed the data, prepared the figures and movies, and wrote the corresponding text. RS-N and MA contributed to writing and editing the manuscript. VF designed and performed the experiments with *His2av::EGFP* and dual-fluorophore labelling of the lamina, supervised the project, and contributed to writing and editing the manuscript. All authors contributed to the article and approved the submitted version.

## Conflict of Interest

The authors declare that the research was conducted in the absence of any commercial or financial relationships that could be construed as a potential conflict of interest.

## Supplementary Material

Supplementary Movie 1 Neuroblast divisions in the third instar brain lobe. Time-lapse 2-photon imaging of wandering L3 (wL3) larval brain lobes. Example neuroblast and GMC divisions are highlighted; neuroblasts were identified by their large cell size. His2Av::eGFP marks chromosomes. Timescale displayed as hh:mm:ss, scale bar = 10 μm.

Supplementary Movie 2 Neuroblasts reactivating from quiescence. Time lapse 2-photon imaging of *grh > syn21-GFP-p10* labeled neuroblasts in late L1 VNC. Explants oriented laterally to best visualize the neuroblast fibers, present during quiescence. The movie starts 24 h after larval hatching, a time when most VNC neuroblasts are quiescent. Reactivation divisions were first observed at movie time 1 h 40 min. Compared with membrane-localized GFP, cytoplasmic GFP requires higher levels of expression to achieve similar brightness in fine cellular processes due to unfavorable surface area-to-volume ratios (Pfeiffer et al., 2012). Timescale displayed as hh:mm:ss, scale bar = 20 μm.

Supplementary Movie 3 Basal fiber inheritance upon neuroblast reactivation. (Part 1) Time lapse imaging of *grh > CD4::tdTomato* neuroblast in late L1 VNC undergoing first post-reactivation division. Movie captures fiber inheritance by the firstborn GMC. Timescale displayed as hh:mm:ss, scale bar = 10 μm. (Part 2) Time lapse imaging of a neuroblast and GMC labeled with *grh > CD8::GFP, syn21-GFP-p10*. The GMC which has inherited the fiber divides, and the fiber is inherited by one of the daughter cells. Time scale displayed as hh:mm:ss, scale bar = 5 μm.

Supplementary Movie 4 Growth of the larval lamina. Time-lapse 2-photon imaging of *E-Cad::GFP* (cyan), *27G05 > mCherry* (magenta) demonstrating lamina growth. Brains were oriented to visualize the lamina in a lateral view. The lamina furrow, photoreceptors, and lamina columns were easily distinguishable using *E-Cad::GFP*; lamina precursors and neurons were labeled with *27G05 > mCherry*. We observed that whilst the lamina increased in width during the course of the movie (∼18 h) the position of the lamina furrow (asterisk) did not move significantly. Timescale displayed as hh:mm:ss, scale bar = 20 μm.

Supplementary Movie 5 Incorporation of lamina precursor cells into lamina columns. Time-lapse 2-photon imaging of gcm > nlsGFP. We saw multiple LPCs from the lamina assembly domain incorporate into columns. The positions of the lamina furrow, lamina pre-assembly domain, photoreceptor axon entry point, lamina columns and epithelial and marginal glia are marked. In the first half of the movie LPCs were tracked (colored dots) and seen to incorporate into multiple columns simultaneously. In the second half, epithelial and marginal glia were tracked (colored dots) with several re-locating to different positions. Note: Dots remain in the last observed position of a cell if we could not track it due to movement out of plane. We also observed the incorporation of an epithelial glia into the youngest lamina column. Timescale displayed as hh:mm:ss, scale bar = 20 μm.

Supplementary Movie 6 Migration of lamina wide-field neurons and epithelial and marginal glia. Time-lapse 2-photon imaging of *gcm > CD8::GFP* (expressed in Lawfs, epithelial and marginal glia and lamina precursors) captured the migration of Lawfs, and epithelial and marginal glia. The explant was oriented such that a cross-section of the lamina could be seen together with the dorsal arm of the lamina crescent. A maximum intensity projection is presented to account for movement in the z dimension. The video starts with Lawf migration marked by colored dots and the second half of the video shows epithelial and marginal glial migration, also marked by colored dots. Note: Dots remain in the last observed position of a cell if we could not track it due to movement out of plane. Timescale displayed as hh:mm:ss, scale bar = 20 μm.

Supplementary Movie 7 Migration of epithelial and marginal glia. Using *dpp > flexamp* to induce stochastic expression of myristoylated-GFP, time-lapse 2-photon imaging was employed to capture eg/mg migration. The brains were oriented such that a lateral view of the lamina could be seen. A maximum intensity projection is presented to account for movement in the z dimension. The lobula plug and glial progenitor domains of the OPC can be seen. Epithelial and marginal glia migrate toward the central region of the lamina (marked with arrows). Epithelial and marginal glia migrating are labeled on the right-hand side of the lamina and their migration path is indicated. A cropped zoom clearly shows filopodia-like projections in migrating epithelial and marginal glia. Timescale displayed as hh:mm:ss, scale bar = 20 μm.

Supplementary Movie 8 Eye-antennal disc eversion in third instar larva. Wandering L3 eye discs (attached to the underlying CNS) ubiquitously expressing membrane bound GFP (*ubi-GFP-CAAX*) to visualize eye-antennal disc eversion. As eversion begins, the peripodial epithelium is seen to contract (indicated by arrows) and pull the eye disc toward the antennal disc which becomes circular in shape. Disc eversion is completed within 5 h. Timescale displayed as hh:mm:ss, scale bar = 40 μm.

Supplementary Movie 9 Cyst stem cell (CySC) division in the adult testis stem cell niche. A time-lapse movie of *tj > CD8::GFP*, which labels the somatic lineage. The hub is indicated by a magenta dot. CySCs are the first row of labeled cells around the hub. The CySC labeled with a blue dot undergoes a division to produce two daughter cells (blue and green dots). The nuclear membrane remains visible throughout mitosis of the CySC. Timescale displayed as hh:mm:ss, scale bar = 10 μm.

Supplementary Movie 10 Nuclear envelope retention during CySC division. A time-lapse movie of *tj > CD8::GFP*, which labels the somatic lineage. The hub is marked by a blue dot, a dividing CySC is marked by a green dot. During mitosis, the labeled CySC rounded up. Once again, the nuclear membrane could be clearly observed during mitosis and adopted a distinctive diamond shape. Note that the division occurs out of plane such that only one daughter is visible after the division. Timescale displayed as hh:mm:ss, scale bar = 20 μm.
